# Clinical outcomes in distal radial fractures with ipsilateral arteriovenous fistulas

**DOI:** 10.1186/s13018-019-1171-4

**Published:** 2019-05-22

**Authors:** Hao-Ming Chang, Yi-Chuan Chou, I-Ming Jou, Jui-Ming Yang, Ching-Hou Ma, Po-Ting Wu

**Affiliations:** 1 Department of Orthopedics, National Cheng Kung University Hospital, College of Medicine, National Cheng Kung University, Tainan, 701 Taiwan; 2 Medical Device R & D Core Laboratory, National Cheng Kung University Hospital, Tainan, Taiwan; 3 Department of Orthopedics, Tainan Hospital, Ministry of Health and Welfare, Tainan, Taiwan; 4 Department of Orthopedics, E-Da Hospital, Kaohsiung, Taiwan; 5 School of Medicine, College of Medicine, I-Shou University, Kaohsiung, Taiwan; 6 Department of Orthopedic, Tainan Sin Lau Hospital, Tainan, Taiwan; 7 Department of Orthopedics, College of Medicine, National Cheng Kung University, Tainan, Taiwan; 8 Department of Biomedical Engineering, National Cheng Kung University, Tainan, Taiwan; 9 Department of Orthopedics, National Cheng Kung University Hospital Dou-Liou Branch, College of Medicine, National Cheng Kung University, Dou-Liou, Taiwan

**Keywords:** Arteriovenous fistula, Volar locking plate, External fixation, Henry approach, Distal radius fracture, Hemodialysis patients

## Abstract

**Background:**

We evaluated the effects on arteriovenous fistula (AVF) function and clinical outcomes in patients given cast fixation, external skeletal fixation [ESF], or volar locking plate fixation [VLPF] for an ipsilateral distal radial fracture (DRF).

**Methods:**

Thirteen patients were assigned to the surgery group or the cast group; follow-up was ≥12 months. One-year clinical outcomes and serial AVF function and radiographic outcomes were recorded and analyzed.

**Results:**

All fractures were union and all AVFs were preserved with continuous hemodialysis. The surgery group had better immediately (radial inclination and articular step-off) and 1-year post-index procedure radiographic findings (radial height, radial inclination, volar tilting, ulnar variance, and articular step-off) and better 1-year functional outcomes (Mayo and *Quick*DASH score) than did the cast group. The VLPF subgroup had better *Quick*DASH scores and radiographic outcomes (radial inclination and ulnar variance) than did the ESF subgroup.

**Conclusions:**

One year after the index procedure, none of the treatment affected shunt function in DRFs ipsilateral to AVFs. ESF and VLPF yielded better functional and radiographic outcomes than did cast fixation in patients with ipsilateral DRFs and AVFs.

**Level of Evidence:**

III

## Background

Upper extremity fractures ipsilateral to an arteriovenous fistula (AVF) in hemodialysis patients are not rare [1]. Surgical treatment options are still challenging and controversial because of the danger of hypervascularity, hemorrhaging, contraindications of using a pneumatic tourniquet [1], remodeled anatomy after shunt creation, and the potential effects of the type of treatment on the function of arteriovenous shunts. Only few case series have reported the clinical outcomes of surgical repair for distal radial fracture (DRF) ipsilateral to an AVF [1, 2].

In our clinical practice, in addition to cast fixation, surgical repairs—volar locking plate fixation (VLPF) and external skeletal fixation (ESF)—are also feasible treatment options. Our search of the literature showed that no published study has compared the effects of VLPF, cast fixation, or ESF on ipsilateral AVF function. Therefore, we evaluated the effects of VLPF, cast fixation, and ESF on ipsilateral AVF function and clinical outcomes. We hypothesized that none of these treatments would affect the AVF function after the AVF had been carefully identified and the fracture was carefully managed before and during the procedure. We also hypothesized that surgical treatment would provide better clinical and radiographic outcomes in this population.

## Materials and methods

We retrospectively reviewed the medical records of hemodialysis patients at our hospital who had had an isolated DRF between June 2007 and May 2016. The study protocol was approved by our Institutional Review Board.

### Patients

We identified 13 patients (12 women and 1 man; mean age 65 years; age range 44–78 years) who had been followed-up for at least 12 months. The inclusion criteria were (1) an isolated DRF ipsilateral to an AVF, (2) having been treated with VLPF, ESF, or cast fixation, or (3) having been followed-up for at least 12 months post-surgery. The exclusion criteria were (1) AVF placement for less than 6 months, (2) an incomplete medical record, (3) a pre-existing DRF, and (4) other fractures in addition to a DRF.

Treatment included cast fixation, ESF (DepuySynthes External Distal Radius Fixator, Swiss), and VLPF (Aplus® Distal Radius Locking Plate System; Aplus, Taipei, Taiwan). All patients received the close reduction and long arm cast first. If the primary reduction was unacceptable: dorsal radial tilt > 10°, radial shortening > 3 mm, and any intraarticular step-off > 2 mm [3], surgical intervention was suggested. The final surgical treatment choice (ESF or VLPF) was determined after a discussion with the patient. All AVFs were detected using palpation of thrills and auscultation of bruits. In cases with obscure physical examination, preoperative color Doppler ultrasonography would be applied to confirm the location of AVF.

**Fig. 1:**
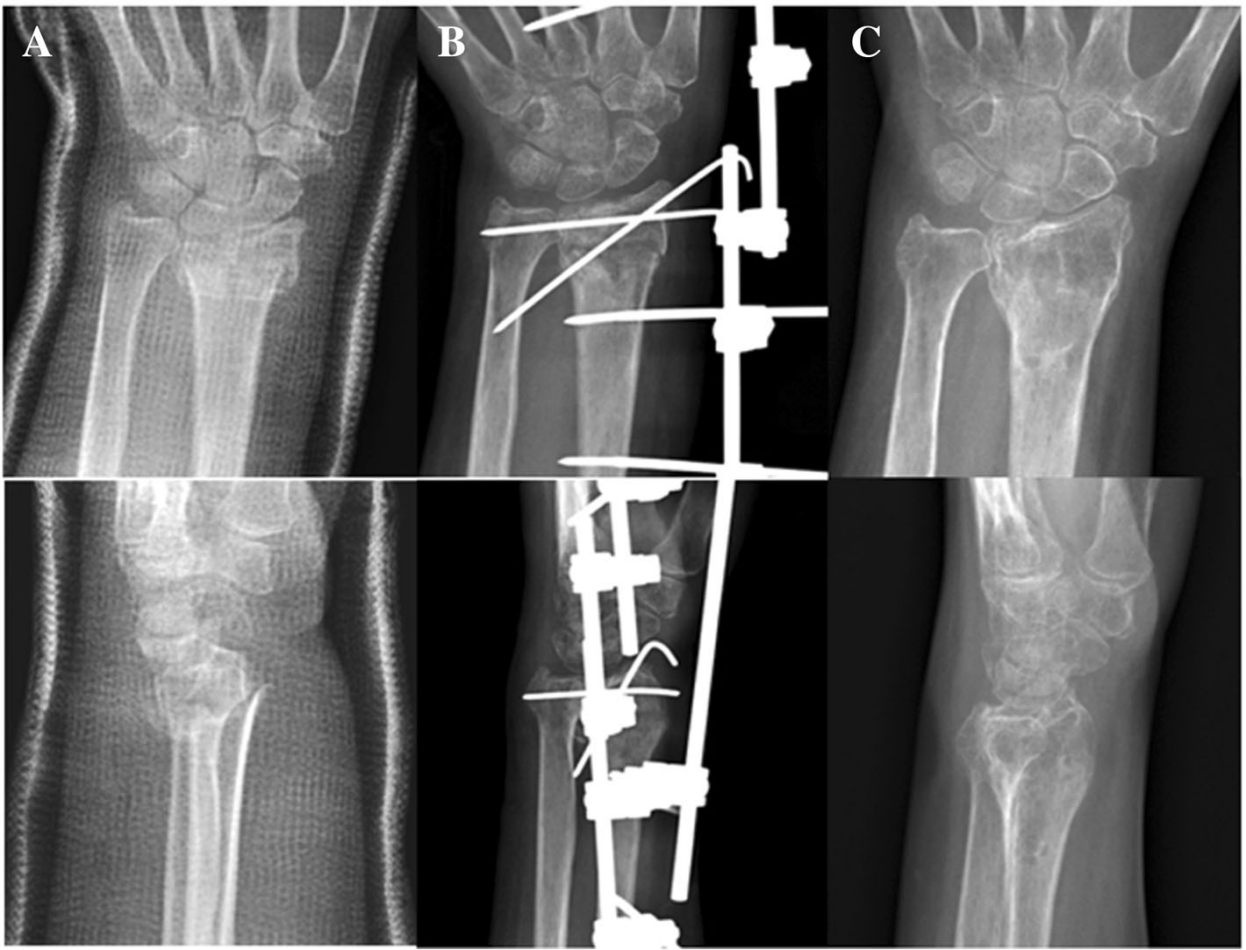
The 67-year-old female with right distal radius fracture (AO type C2) and the ipsilateral radiocephalic shunt was treated with external skeletal fixation and K-wires. The serial images are preoperative (**a**), immediately postoperative (**b**), and 1-year follow-up (**c**) radiographs. The upper row was the anteroposterior view and the lower row was the lateral view

**Fig. 2:**
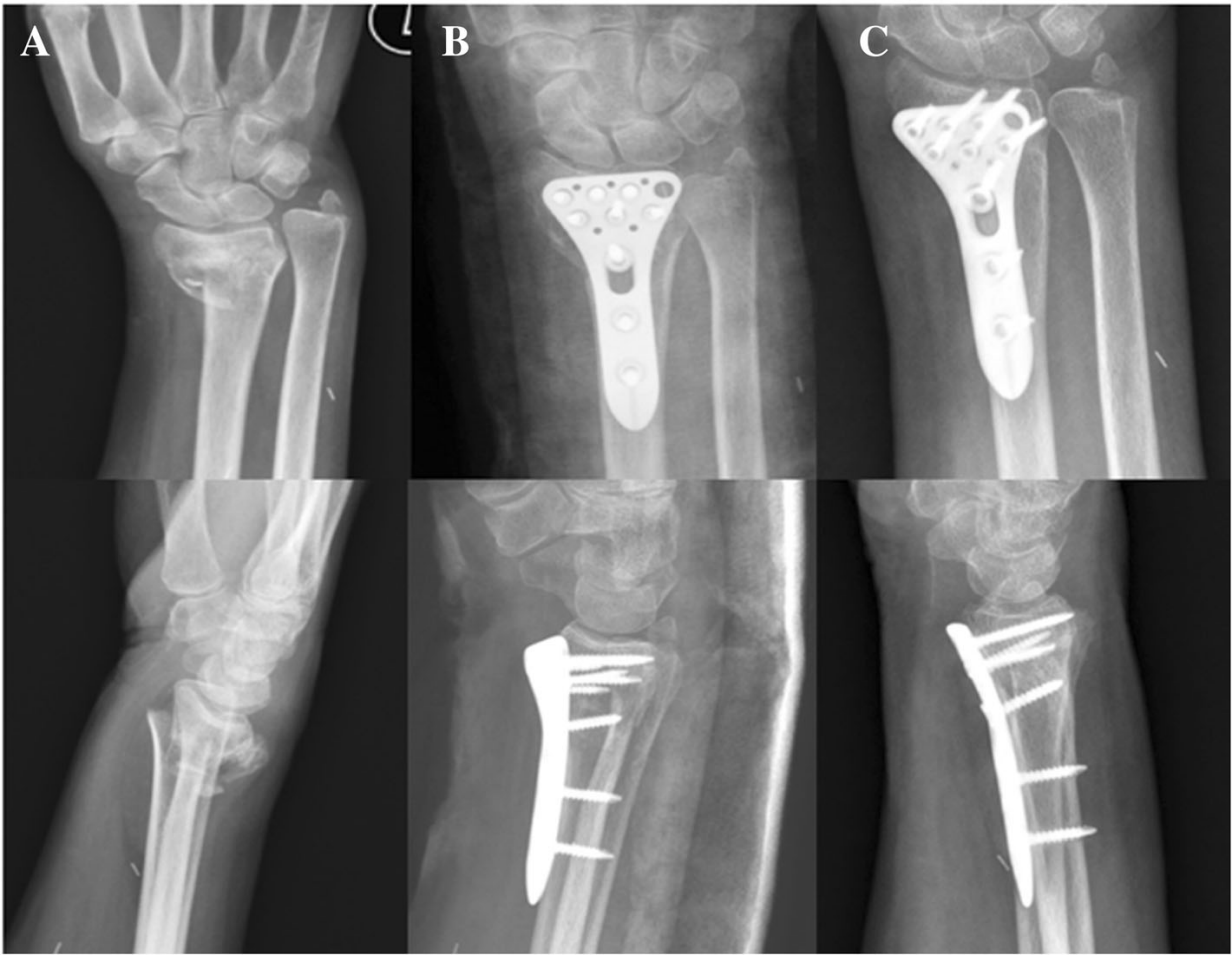
The 67-year-old female with left distal radius fracture (AO type C2) and the ipsilateral brachiocephalic shunt was treated with a volar locking plate. The serial images are preoperative (**a**), immediately postoperative (**b**), and 1-year follow-up (**c**) radiographs. The upper row was the anteroposterior view and the lower row was the lateral view

Surgical intervention (ESF and VLPF) was done under general anesthesia without a tourniquet. Close reduction and fixation with percutaneous K-wire and ESF was done using a C-arm fluoroscope (Fig. 1). Open VLPF reduction was done using a modified Henry approach (Fig. 2). Electrocoagulation was used to control intraoperative bleeding and hemostasis. The mini-hemovac drainage was placed in the VLPF group and removed on the next day. Bone grafts were not applied in our cases. Casting with close reduction was done based on finger-trap traction and manual pressure [4] using an adequate hematoma block [5]. We created on each cast a window of appropriate size for hemodialysis. Above-the-elbow casting was used for the initial 4 weeks, and below-the-elbow cast for the subsequent 2 weeks, after which the cast was removed.

Regular hemodialysis with heparin and normal saline rinse was done 1 day after surgery using the patient’s original hemodialysis schedule. We analyzed pre-procedure (p), immediately post-index procedure (i), and 1-year post-index procedure (f) radiographic outcomes using five parameters: radial height [RH], radial inclination [RI], volar tilting [VT], ulnar variance [UV], and step-off [SO]. We also analyzed hand (QuickDASH) scores [6], and modified Mayo wrist scores [7], from the last visit or chart record, and AVF function based on the 2006 National Kidney Foundation Kidney Disease Outcomes Quality Initiative (NKF-K/DOQI) guidelines [8].

### Statistical analysis

Because of our small sample of patients, we used the Mann-Whitney test for between-group comparisons of VAS, the parameters of radiographic findings, clinical functional outcomes, and AVF functions. All data for variables were expressed as medium (Q1, Q3). Significance was set at *P* < 0.05.

## Results

### Patient demographic data

All 13 patients had been injured in a simple fall. Twelve of the fractures were in the non-dominant limb, and one was in the dominant. All fractures were intraarticular and, based on the AO/OTA Classification of Fractures and Dislocations (formerly the Müller/AO Classification), were classified as partially articular type B (B2 in two cases, B3 in one case) or completely articular type C (C1 in two cases, C2 in seven cases, and C3 in one case). Four patients had undergone VLPF; four, ESF; and the other five, cast fixation. Six patients had brachiocephalic shunts, and seven had radiocephalic shunts (Table 1). At the follow-up, all AVFs were preserved, and adequate hemodialysis was achieved using a urea reduction ratio (URR) > 65%, and a Kt/V > 1.2 (*K*, dialyzer clearance [mL/min]; *t*, time [s]; *V*, volume of water a patient’s body contains) [8].

**Table 1:** Demographic data

No.	Sex	Age at injury (years)	Fracture pattern (AO/OTA classification)	Follow-up (months)	Arteriovenous fistula type
Cast = 5					
1	F	66	C1	13	B-C
2	M	75	C1	12	B-C
3	F	53	C3	12.5	R-C
4	F	65	C2	13.5	B-C
5	F	62	B2	12	B-C
External skeletal fixation (ESF) = 4					
6	F	44	C2	12	R-C
7	F	73	C2	13	R-C
8	F	64	B2	12	R-C
9	F	67	C2	12	R-C
Volar locking plate fixation (VLPF) = 4					
10	F	61	C2	14	R-C
11	F	69	B3	12	B-C
12	F	67	C2	13.5	B-C
13	F	78	C2	13.5	R-C

*F* female, *M* male, *R-C* radiocephalic shunt, *B-C* brachiocephalic shunt

### Surgery vs. cast group

The surgery group had significantly (*P* < 0.05) better functional scores (Mayo wrist score and *Quick*DASH score) than did the cast fixation group (Table 2). A radiographic analysis showed no significant difference in preoperative radiographic parameters. The surgery group had significantly (*P* < 0.05) better RI and SO immediately post-index procedure and all radiographic parameters (RH, RI, VT, UV, and SO) at the 1-year follow-up (Table 3).

**Table 2:** Postoperative functional scores in surgical (external fixator and volar locking plate fixation) and cast fixation groups at 1 year after the index procedure

Variable	Surgical fixation	Cast fixation	*P*
VAS (score 1–10)	3.0 (1.0, 3.8)	4.0 (3.5, 5.0)	0.065
Mayo score (score 1–100)	62.5 (60.0, 76.3)	40 (40.0,57.5)	0.030*
*Quick*DASH (score 0–100)	18.0 (6.9, 31.2)	50 (46.5, 68.75)	0.002*

All data for variables were expressed as medium (Q1, Q3)

*ESF* external skeletal fixation, *VLPF* volar locking plate fixation, *VAS* visual analog scale

*Significantly different (*P* < 0.05) in Mann-Whitney test

**Table 3:** Preoperative and postoperative radiographic findings in surgical (external skeletal fixation and volar locking plate fixation) and cast fixation groups

Variable	Cast fixation	Surgical fixation	*P*
pRH	5.2 (4.0, 9.9)	8.4 (6.9, 9.8)	0.354
pRI	13.3 (9.5, 19.6)	20.0 (17.1, 22.9)	0.222
pVT	− 16 (− 23, − 10)	− 25.2 (− 29.4, − 19.8)	0.093
pUV	3.72 (2.9, 4.1)	4.6 (2.3, 8.1)	0.435
pSO	2.4 (1.3, 3.0)	2.0 (1.4, 2.2)	0.354
iRH	11.2 (10.8, 11.9)	11.5 (10.9, 12.2)	0.833
iRI	13.3 (11.4, 15.6)	20.9 (19.8, 24.3)	0.002*
iVT	− 2.6 (− 5.5, − 1.2)	7.5 (− 2.3, 9.5)	0.093
iUV	1.9 (1.1, 2.3)	0.15 (0, 1.9)	0.127
iSO	1.8 (1.4,1.9)	0.4 (0.3, 0.7)	0.030*
fRH	4.9 (− 0.9, 6.2)	10.2 (9.4, 11.4)	0.003*
fRI	10.6 (− 0.3, 14.5)	20.0 (15.4, 23.7)	0.030*
fVT	− 16 (− 26.5, − 12.5)	4.1 (− 7.3, 10.8)	0.002*
fUV	3.7 (2.95, 8.2)	1.4 (0.2, 2.4)	0.019*
fSO	2.8 (1.67, 3.95)	0.4 (0.2, 1.1)	0.006*

All data for radiological variables were expressed as medium (Q1, Q3) and units for all data were in millimeters (mm)

*RH* radial height, *RI* radial inclination, *VT* volar tilt, *UV* ulnar variance, *SO* step-off, *p* pre-procedure, *i* immediate post-procedure, *f* final follow-up (1 year after the index procedure)

*Significantly different (*P* < 0.05) in Mann-Whitney test

### ESF vs. VLPF

The VLPF group had a significantly (*P* < 0.05) better QuickDASH score than did the ESF group (Table 4). There were no significant differences in VAS score and Mayo wrist score. A radiographic analysis showed that the VLPF group had a significantly (*P* < 0.05) better UV at immediately postoperatively phase and better UV and RI at the 1-year follow-up than did the ESF group (Table 5). There were no wound infections, pin infections, or other complication in our study.

**Table 4:** Postoperative functional scores in external skeletal fixation (ESF) and volar locking plate fixation (VLPF) groups at 1 year after the index procedure

Variable	ESF	VLPF	*P*
VAS (score 1–10)	3.5 (3.0, 4.8)	1.0 (0.3, 2.5)	0.057
Mayo score (score 1–100)	60 (60.0, 63.8)	72.5 (61.3, 83.8)	0.200
*Quick*DASH (score 0–100)	27.5 (20.0, 38.8)	8.8 (4.6, 15.1)	0.029*

All data for variables were expressed as medium (Q1, Q3)

*ESF* external skeletal fixation, *VLPF* volar locking plate fixation, *VAS* visual analog scale

*Significantly different (*P* < 0.05) in Mann-Whitney test

**Table 5:** Preoperative and postoperative radiographic findings in external skeletal fixation (ESF) and volar locking plate fixation (VLPF) groups

Variable	ESF	VLPF	*P*
pRH	8.4 (7.2, 9.6)	8.2 (1.8, 15.3)	1.000
pRI	18.6 (17.1, 21.8)	21.5 (5.0, 31.8)	0.686
pVT	− 25.2 (− 27.1, − 22.7)	− 24.4 (− 51.5, − 13.6)	1.000
pUV	6.39 (2.54, 9.3)	4.1 (0.9, 6.8)	0.486
pSO	2.2 (2.0, 2.4)	1.6 (1.2, 2.0)	0.057
iRH	10.9 (10.4, 13.9)	12.0 (11.2, 12.2)	0.343
iRI	21.0 (19.8, 26.5)	20.9 (17.9, 24.1)	0.886
iVT	4.0 (− 6.0, 9.5)	7.5 (− 0.5, 10.3)	0.686
iUV	1.9 (0.7, 2.2)	0.0 (− 1.3,0.0)	0.029*
iSO	0.5 (0.3, 1.8)	0.4 (0.3, 0.6)	0.886
fRH	10.2 (7.5, 11.2)	10.3 (9.4, 12.5)	0.886
fRI	16.7 (7.0, 19.5)	22.8 (20.6, 25.6)	0.029*
fVT	− 3.4 (− 11.0, 8.5)	8.5 (− 2.0, 16.0)	0.343
fUV	2.3 (1.9, 4.2)	0.3 (0.1, 0.8)	0.029*
fSO	0.7 (0.2, 1.7)	0.4 (0.3,0.6)	1.000

All data for radiological variables were expressed as medium (Q1, Q3) and units for all data were in millimeters (mm)

*RH* radial height, *RI* radial inclination, *VT* volar tilt, *UV* ulnar variance, *SO* step-off, *p* pre-procedure, *i* immediate post-procedure, *f* final follow-up (1 year after the index procedure)

*Significantly different (*P* < 0.05) in Mann-Whitney test

## Discussion

This is the first study to compare functional and radiographic outcomes in different treatments of DRFs ipsilateral to AVFs. All DRFs in our study were intraarticular, and all AVFs were preserved and provided adequate hemodialysis at 1 year after the index procedure. At 1-year follow-up, the surgical fixation groups (ESF and VLPF) had better functional (Mayo score and *Quick*DASH score) and all radiographic (RH, RI, VT, UV, and SO) outcomes than did the cast fixation group. Furthermore, VLPF had better *Quick*DASH scores and radiographic RI and UV than did the ESF group.

A DRF ipsilateral to an AVF is not rare, but there are scant studies on this condition. There are several treatment choices for managing a DRF, including cast fixation, ESF, and VLPF [9]. When managing a DRF ipsilateral to an AVF, most patients and surgeons hesitate to use surgical intervention because of the dangers of hypervascularity, hemorrhaging, and contraindications to using a pneumatic tourniquet [1]. Therefore, closed reduction with cast fixation and an open window for hemodialysis became an alternative treatment. However, the fracture pattern in this population is usually intraarticular and unstable [10] (in our series 13/13), because of osteomalacia and hemodialysis-related osteoporosis. In our cast fixation group, even acceptable reduction [3] was achieved after close reduction in the initial attempt, and it is difficult to maintain the reduction during the follow-up. Another possible reason is that the hemodialysis window in the cast diminishes the effect of cast fixation. Other possibilities are pressure ulcers, compartment syndrome, dermatitis, and joint contracture caused by cast fixation [11]. Therefore, surgical fixation is becoming more popular. Sugiyama et al. [2] reported that three patients with a DRF ipsilateral to an AVF who underwent VLPF using the Henry approach had satisfactory alignment without shunt complications. Ishiguro et al. [1] reported that one patient with a Colles’ fracture ipsilateral to an AVF who underwent cement-assisted balloon osteoplasty had a satisfactory outcome without shunt complications.

In our series, the surgical fixation group showed better final radiographic RH, RI, VT, UV, and SO findings than did the cast fixation group, which is consistent with studies that showed better radiographic outcomes in the elderly using surgical intervention rather than cast fixation, the conservative treatment [12, 13]. Even if the acceptable reduction [3] could be achieved in the initial reduction in the cast fixation group, the surgical fixation group still showed better immediately postoperative radiographic RI and SO. Correspondingly, at the final 1-year follow-up, the cast fixation group also showed significantly worse functional outcomes: Mayo score and *Quick*DASH score. Our results are inconsistent with the study that reported no differences in functional outcomes cast and surgical fixation [14]. Therefore, according to our findings, surgical fixation should be considered in an unstable DRF [10] ipsilateral to an AVF, even with the initial acceptable reduction.

In our study, surgical fixation did not affect AVF function, which supports the findings of the previous study [2]. Furthermore, VLPF provided better UV radiographic outcome immediately after surgery, better RI and UV at the 1-year follow-up, and better *Quick*DASH scores at the 1-year follow-up than did ESF. There were no differences in the VAS or Mayo scores between the ESF and VLPF groups at the 1-year follow-up. In the general population, recent meta-analyses have reported better specific radiographic and functional outcomes and a faster recovery with VLPF than with ESF [15, 16]. In an elderly population, compared with ESF, VLPF provided earlier functional recovery [17, 18], better restoration of palmar tilt, and better VAS, wrist function, and DASH scores after the final follow-up [19]. Therefore, VLPF might be a better fixation choice than ESF for hemodialysis patients with DRFs and ipsilateral AVFs. However, hemodialysis patients usually spend a considerable amount of money on hemodialysis. ESF might be an alternative if patients with an unstable DRF cannot afford VLPF treatment cost.

Up to date, there is no study addressing the relationship between the tourniquet time and the possible complications on AVFs, including thrombosis formation and further stenosis. Even though Naito et al. [20] reported no complication of AVFs following carpal tunnel release using a pneumatic tourniquet in patients with chronic renal dialysis, the application of tourniquet in patients with DRFs and ipsilateral AVFs is still a concern. Therefore, hemorrhaging might be expected because of uremic platelet dysfunction, especially when a tourniquet was not used, but hemorrhages can be controlled using adequate electrocoagulation. Furthermore, shunt and the pulsation of the radial artery that are very easy to be palpated in the absence of tourniquet could be safely checked during the surgery, especially in the application of K-wires or Schanz screws. Hence, we suggest meticulous hemostasis without application of tourniquet in patients with DRFs and ipsilateral AVFs. The radial artery runs between the brachioradialis and flexor radialis tendons. The modified Henry approach that avoids the identification of the radial artery may avoid the potential injury of the radial artery. The AVF is usually more on the radial side of the forearm than is the radial artery. Sugiyama et al reported three patients with DRFs ipsilateral to AVFs treated with volar locking plate fixation via the Henry approach achieved good result and no shunt dysfunction [2]. According to our results, there is no AVF complication in all four cases undergoing VLPF using a modified Henry approach. Both the Henry and the modified Henry approach may be safe for VLPF in patients with DRFs and ipsilateral AVFs. The reduction techniques used in our cases—provisional K-wire fixation, radial styloid pinning through the snuffbox approximately to the radiocephalic fistula, and the Kapandji technique—were safe for blood vessels monitored using a C-arm fluoroscope and palpable thrill. In addition, anatomical remodeling after shunt creation should also be considered. For ESF and VLPF, overshooting of drilling or longer screw position to the dorsal-radial side is dangerous and should be avoided. For ESF, we practiced predrilling through the sleeve to protect soft tissue and using self-drilling Schanz screws, which should be inserted directly to the bone with gentle retraction around the tissue to ensure that no vessel will be damaged.

Diminishment and failure of AVFs were the most concerned issues during postoperative care in patients with AVFs following ipsilateral surgical interventions. Up to date, there is no literature reporting postoperative complications of AVFs in such population. Some most common complication of AVFs had been mentioned in some literatures and could probably be as a reference for postoperative care [21–23]. Thrombosis is one of the crucial causes for the loss of function of an AVF. The clinical feature of thrombosis included severe pain at the site of thrombosis, palpation of thrombus at the AVF site, tremors, and absence of feeling [23]. In addition, AVF infection may be manifested as local signs of infection (calor, dolor, and rubor) [23]. Hence, in addition to general postoperative care of distal radial fracture, the appearance and auscultation of an AVF and the related hemodialysis condition should be closely observed after the index procedure.

### Limitations

This study has some limitations. First, our study population is small after excluding patients with incomplete records. However, the differences between our comparison groups were significant, which suggests that the number of patients is adequate to test our hypothesis. Second, a treatment selection bias existed depending upon the attending surgeon, patient selection, and the patient’s economic status. Third, we used *Quick*DASH and the Mayo wrist score as functional scores to assess the hemodialysis hand—usually the non-dominant hand. Many activities might be compensated by the dominant or healthy hand, which would lead to a score assessment bias. However, there are no specific or modified standard methods for assessing AVF hand function.

## Conclusions

We found that at 1 year after the index procedure, neither cast fixation nor the surgical interventions with ESF or VLPF affected shunt function in a DRF ipsilateral to an AVF. In addition, surgical fixation with ESF and VLPF yielded better functional outcomes, including Mayo score and *Quick*DASH score, and better all radiographic RH, RI, VT, UV, and SO than did cast fixation at final follow-up. Therefore, we recommend that the indication for surgical treatment of a dialysis patient with a DRF ipsilateral to an AVF should be the same as for the general population, especially for an unstable DRF.

## Abbreviations

AVF: Arteriovenous fistula
DRF: Distal radial fracture
ESF: External skeletal fixation
RH: Radial height
RI: Radial inclination
SO: Articular step-off
URR: Urea reduction ratio
UV: Ulnar variance
VLPF: Vvolar locking plate fixation
VT: Volar tilting
